# Association Between the Fetal‐Type Posterior Cerebral Artery and Hypertensive Thalamic Hemorrhage

**DOI:** 10.1002/brb3.70147

**Published:** 2024-11-07

**Authors:** Tianqiang Pu, Xuemin Zhong, Guohui Jiang, Zhengjun Wu, Shixu He, Yanling Long, Qiuxia Liang, Xionglong Tu, Suqiu Yao, Jian Wang, Mingfang He

**Affiliations:** ^1^ Department of Cerebrovascular Diseases Guangyuan Central Hospital Guangyuan China; ^2^ Department of Neurology Chengdu Second People's Hospital Chengdu China; ^3^ Department of Neurology Affiliated Hospital of North Sichuan Medical College Nanchong China

**Keywords:** fetal‐type posterior cerebral artery, hypertensive thalamic hemorrhage, homocysteine, risk factors

## Abstract

**Introduction:**

This study investigated the association between the fetal‐type posterior cerebral artery (FTP) and hypertensive thalamic hemorrhage (HTH).

**Method:**

Patients with hypertension who met the admission criteria between January 2020 and August 2022 were retrospectively analyzed and divided into the HTH and non‐HTH groups based on the presence or absence of thalamic hemorrhage, respectively. In addition to whether the variable was an FTP, other variables with unbalanced distributions between the two groups were used as matching factors for 1:1 propensity score matching, and other variables that were not used as matching factors were used as covariates for conditional logistic regression.

**Results:**

A total of 722 patients were included in the study, with 115 in the HTH group and 607 in the non‐HTH group. After propensity score matching, 114 patients were included in both groups. The multivariate logistic regression analysis showed that the FTP (*p* = 0.003, odds ratio [OR]: 2.712, 95% confidence interval [CI]: 1.407–5.225) and homocysteine levels (*p* = 0.007; OR: 1.051; 95% CI: 1.014–1.089) were significantly correlated with the occurrence of HTH.

**Conclusion:**

The incidence of FTP is not low, and it is an independent risk factor for HTH. Early recognition and appropriate monitoring are warranted for cases of FTP.

## Graphical abstract

We found that the FTP (*p* = 0.003, odds ratio [[OR]: 2.712, 95% confidence interval [CI]: 1.407‐5.225) and homocysteine levels (*p* = 0.007; OR: 1.051; 95% CI: 1.014‐1.089) were significantly correlated with the occurrence of HTH. Early recognition and appropriate monitoring are warranted for cases of FTP.

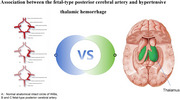

## 1 Introduction

Hypertensive thalamic hemorrhage (HTH) is a common, disabling, and fatal clinical disease that accounts for ∼13% of cerebral hemorrhages (Arboix et al. 2007). Severe neurological function defects can occur because of the deep anatomical location and complex structure of the thalamus, which is close to important neural structures, such as the internal sac, brain stem, hypothalamus, and ventricle (Shah et al. 2005). With an increase in the aging population, the incidence of thalamic hemorrhage is gradually increasing, which is causing a serious burden on families and the society (Neisewander et al. 2018; Teramoto et al. 2017). Therefore, the prevention and treatment of thalamic hemorrhage have been the focus of recent neuroscience research. Recent studies have shown that the prevention and treatment of HTH should focus on controlling blood pressure and that the exposure to other risk factors, such as long‐term smoking, alcoholism, hyperlipidemia, obstructive sleep apnea, obesity, and poor lifestyle, should be avoided (Chen et al. 2015; Chinese Medical Association Branch of Neurosurgery, Chinese Medical Association Branch of Emergency Physicians, Chinese Medical Association Branch of Neurology Group 2020; Chinese Hypertension Prevention Guidelines Revision Committee, Hypertension Alliance (China), Chinese Medical Association Branch of Cardiology 2019).

The fetal‐type posterior cerebral artery (FTP) is a congenital anatomic variant of the circle of Willis, with an incidence of ∼26%. It is believed that most or all of the blood flow in the posterior cerebral artery (PCA) comes from the ipsilateral internal carotid artery (Arjal, Zhu, and Zhou 2014; Frid et al. 2022). With the development of imaging technology, increasing attention has been paid to the relationship between the anatomical variations in the circle of Willis and cerebrovascular diseases. Previous studies have shown that FTP can affect the hemodynamics of collateral compensation of the pia meninges and the anterior and posterior circulation and is correlated with the incidence of cerebrovascular diseases, such as cerebral infarction and aneurysmal subarachnoid hemorrhage (Arjal, Zhu, and Zhou 2014; Dharmasaroja, Uransilp, and Piyabhan 2019; H. J. Lee et al. 2021). The blood supply to the thalamus mainly originates from the basilar artery, PCA, and posterior communicating artery (PCOA; Goto et al. 1982; Tokgoz et al. 2013). FTP changes the anatomical structure of the circle of Willis and the mode of blood circulation. To date, no studies have reported a correlation between FTP and HTH. Therefore, this study aimed to explore the relationship between FTP and HTH and to establish novel ideas for the prevention and treatment of HTH.

## 2 Materials and Methods

### 2.1 General Data Collection

Patients diagnosed with hypertension who were admitted to the Department of Neurology of Guangyuan Central Hospital and Chengdu Second People's Hospital between January 2020 and August 2022 were retrospectively analyzed. The following information was collected: name, sex, and age; cerebrovascular disease risk factors, such as smoking (current or previous smoker within the last 6 months), drinking (intake of more than 80 g/day or previous drinking habits within the last 6 months prior), hypertension (receiving anti‐hypertensive treatment or blood pressure ≥ 140/90 mmHg on repeated measurements), and diabetes (receiving medicine for diabetes mellitus, fasting blood glucose ≥ 7.0 mmol/L, or 2‐h postprandial blood glucose or random blood glucose ≥ 11.1 mmol/L); total cholesterol, triglycerides, high‐density lipoprotein, low‐density lipoprotein, and homocysteine; and whether the patients had an FTP. This study was reviewed and approved by the Ethics Committee of Guangyuan Central Hospital.

### 2.2 Inclusion Criteria and Exclusion Criteria

The inclusion criteria were as follows: (1) diagnosis of essential hypertension based on the diagnostic criteria in the Chinese Hypertension Prevention and Treatment Guidelines (Chinese Hypertension Prevention Guidelines Revision Committee, Hypertension Alliance (China), Chinese Medical Association Branch of Cardiology 2019) and (2) improved CTA examination of the head and neck.

The exclusion criteria were as follows: (1) hemorrhage outside the thalamus or subarachnoid hemorrhage, (2) complications of ischemic stroke or transient cerebral ischemic attack, (3) intracranial and extracranial arterial occlusion, (4) brain tumor, (5) cerebrovascular malformations or vasculitis, (6) coagulation system diseases, (7) cerebral venous sinus thrombosis, and (8) incomplete medical records.

The diagnostic criterion, which was based on the Chinese Multidisciplinary Diagnosis and Treatment Guidelines for Hypertensive Cerebral Hemorrhage (2020 edition), used for FTP is the presence of a communication artery after the internal carotid artery that directly continues into the ipsilateral PCA (Chinese Medical Association Branch of Neurosurgery, Chinese Medical Association Branch of Emergency Physicians, Chinese Medical Association Branch of Neurology Group 2020).

### 2.3 Statistical Processing

The R 4.4.0 software was used for data processing and statistical analyses. In addition to the variable of whether it was FTP, other variables with unbalanced distributions between the two groups were used as matching factors for propensity score matching, and matching accuracy was set to 0.2. The matching algorithm adopted the nearest approach with a matching ratio of 1:1. Measurement data were represented by median and interquartile interval, and the Mann–Whitney *U* test was used to compare the differences between the groups. Counting data were represented by frequency and component ratio, and the Chi‐square test was used to compare the differences between the groups. The status of the FTP was used as an independent variable, the status of thalamic hemorrhage was used as a dependent variable, and other variables that were not used as matching factors were used as covariates for conditional logistic regression. The test level was set at *α* = 0.05.

## 3 Results

### 3.1 General Baseline Data and Results of the Single Factor Analysis

Of the total 722 patients included in the study, 115 were in the HTH group and 607 were in the non‐HTH group. There were 54 cases of HTH combined with FTP and 151 cases of non‐HTH combined with FTP. The HTH and non‐HTH groups were matched at a 1:1 ratio, which led to a total of 228 patients (133 men and 95 women) enrolled in the study. The mean age of the HTH and non‐HTH groups were 45 and 50 years, respectively (Table 1).

**TABLE 1:** General information of patients and results of the single factor analysis affecting HTH.

Variables		Before PSM			After PSM		
		Without HTH	With HTH	*p* value	Without HTH	With HTH	*p* value
Sex (%)	M	328 (54.0)	69 (60.0)	0.282	64 (56.1)	69 (60.5)	0.591
	F	279 (46.0)	46 (40.0)		50 (43.9)	45 (39.5)	
Age (years)		66.00 (56.00, 73.00)	67.00 (58.00, 72.50)	0.450	66.00 (55.00, 74.75)	66.50 (58.00, 72.00)	0.591
Smoking (%)	No	436 (71.8)	65 (56.5)	0.002	66 (57.9)	65 (57.0)	1.000
	Yes	171 (28.2)	50 (43.5)		48 (42.1)	49 (43.0)	
Drinking (%)	No	495 (81.5)	82 (71.3)	0.017	82 (71.9)	82 (71.9)	1.000
	Yes	112 (18.5)	33 (28.7)		48 (42.1)	49 (43.0)	
Diabetes (%)	No	471 (77.6)	95 (82.6)	0.283	94 (82.5)	94 (82.5)	1.000
	Yes	136 (22.4)	20 (17.4)		48 (42.1)	49 (43.0)	
Total cholesterol (mmol/L)		4.38 (3.79, 5.15)	4.48 (3.79, 5.01)	0.843	4.38 (3.75, 5.20)	4.46 (3.79, 5.01)	0.824
Triglyceride (mmol/L)		1.40 (0.99, 2.05)	1.15 (0.82, 1.53)	0.001	1.17 (0.84, 1.69)	1.15 (0.82, 1.53)	0.649
High‐density lipoprotein (mmol/L)		1.23 (1.05, 1.46)	1.40 (1.16, 1.58)	0.001	1.35 (1.18, 1.60)	1.40 (1.16, 1.57)	0.810
Low‐density lipoprotein (mmol/L)		2.46 (1.96, 3.02)	2.40 (1.90, 2.96)	0.590	2.46 (1.82, 2.91)	2.40 (1.89, 2.97)	0.654
Homocysteine (µmol/L)		14.50 (11.05, 18.80)	15.40 (11.70, 21.65)	0.067	13.45 (10.72, 16.98)	15.45 (11.65, 21.67)	0.014

*Note*: Values are presented as median (interquartile interval).

Abbreviations: HTH, hypertensive thalamic hemorrhage; PSM, propensity score matching.

### 3.2 Multivariate Logistic Regression Analysis

**FIGURE 1:**
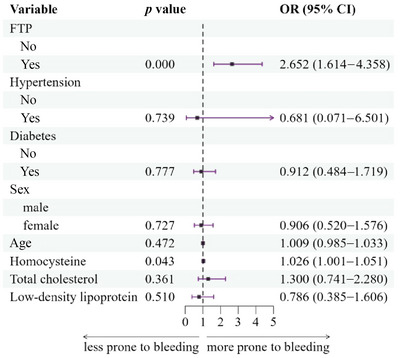
Forest map of the risk factors affecting hypertensive thalamic hemorrhage. CI, confidence interval; FTP, fetal‐type posterior cerebral artery; OR, odds ratio.

The multivariate logistic regression analysis showed that FTP (*p* = 0.003, odds ratio [OR]: 2.712, 95% confidence interval [CI]: 1.407–5.225) and homocysteine (*p* = 0.007, OR:1.051, 95% CI:1.014–1.089) levels were significantly correlated with the occurrence of HTH (Figure 1).

## 4 Discussion

During the early stages of embryonic development, the PCOA gradually develops into the PCA. During brain development, the PCOA gradually thins. The diameter of the basilar artery gradually increases and eventually replaces the PCOA to become the main blood‐supplying artery in the PCA (Dharmasaroja, Uransilp, and Piyabhan 2019). When the P1 segment of the PCA is abnormal or absent, the internal carotid artery system becomes the main blood supply artery of the PCA, forming the FTP (R. M. Lee 1995). In this study, there were 722 patients with HTH, including 205 (28%) patients with FTP and 151 patients in the non‐HTH group, which was consistent with the incidence of FTP reported by Arjal, Zhu, and Zhou (2014), which was ∼26%.

We found that the FTP was significantly associated with HTH. The blood‐supplying arteries of the thalamus mainly include the arteries of the tuberothalamic artery, the arteries of the thalamic geniculate body, the perforating arteries of the thalamus, and the posterior choroidal arteries (Schmahmann 2003), and the above perforating arteries emanate from the PCA almost at a right angle. Under the effect of long‐term hypertension, these perforating arteries are prone to atherosclerosis and rupture and bleeding (Chung et al. 1996; Teramoto et al. 2017). Hademenos and Massoud (1997) found that the structure of bifurcated vessels is prone to causing local blood flow disorders and eddy currents, resulting in hemodynamic changes and susceptibility to atherosclerosis, whereas FTP emanates from the internal carotid artery at an angle; thus, the perforator thalamic artery is prone to atherosclerosis. In the case of unilateral or bilateral FTP, compensatory blood flow in the ipsilateral internal carotid artery system increases (Roman‐Filip et al. 2021), and the blood flow velocity in the internal carotid artery system increases significantly (Jia et al. 2022; Silva Neto, Câmara, and Valença 2012). Cerebral perfusion imaging studies have also confirmed that unilateral FTP can lead to increased blood flow in the blood supply area of the PCA, resulting in similar hyperperfusion manifestations (Wentland et al. 2010). Under a long‐term high load on the internal carotid artery system, the perforator thalamic artery is prone to injury and degeneration (Ryu and Kim 2022). During unilateral FTP, the contralateral PCA accepts the blood flow of the entire vertebrobasilar artery system, which increases the blood flow load of the contralateral PCA and easily leads to injury and degeneration of the perforator artery of the PCA that then leads to atherosclerosis (Meng, Li, and Li 2014). The abnormal blood flow channels and hemodynamic changes caused by FTP are likely to cause atherosclerosis of the perforator thalamic artery, which is the primary cause of HTH, which may explain the effect of FTP on the incidence of HTH.

Our study also found that homocysteine levels were significantly associated with the occurrence of HTH. Homocysteine is a sulfur‐containing non‐protein amino acid and an intermediate product of methionine metabolism (Jakubowski 2017). Some animal experimental studies have shown that blood pressure increases alongside an increase in homocysteine levels (Rolland et al. 1995; Shirpoor et al. 2013). Homocysteine can cause hypertrophy of vascular endothelial cells, hyperplasia and hypertrophy of smooth muscle cells in the middle layer, and migration to the endothelium, resulting in increased collagen fibers in the interstitium and increased stiffness of the vascular wall. These vascular lesions are very similar to those observed in hypertensive cerebral hemorrhage, suggesting a close relationship between homocysteine and hypertensive cerebral hemorrhage (Araki et al. 1989; Rolland et al. 1995; Sharabi et al. 1999; Shirpoor et al. 2013). Previous clinical retrospective case studies found that homocysteine levels were significantly increased in patients with essential hypertension complicated by intracerebral hemorrhage, suggesting that an increase in plasma homocysteine had an effect on essential hypertensive intracerebral hemorrhage (Chang et al. 2015; Qin and Zhang 2003).

This study has some limitations. First, we included samples from two hospitals in this retrospective study. Although the sample size was relatively large, certain problems may have still been evident, such as sampling errors and selection bias. Second, we failed to discuss the impact of FTP classification on HTH. Therefore, the relationship between FTP and HTH warrants further investigation in large prospective population–based cohort studies.

## 5 Conclusions

Our study revealed that FTP was an independent risk factor for HTH. In clinical practice, close observation should be paid to cerebrovascular screening for patients with hypertension. It has been found that patients with FTP variations and cerebrovascular disease risk factors are additive, which increases the possibility of thalamic hemorrhage. Active treatment and control of other risk factors should be performed as soon as possible to prevent the occurrence of HTH.

## Author Contributions

**Tianqiang Pu**: conceptualization, methodology, writing–original draft, writing–review and editing, visualization, formal analysis, resources. **Xuemin Zhong**: writing–original draft, writing–review and editing, formal analysis, conceptualization, methodology, resources, visualization. **Guohui Jiang**: funding acquisition. **Zhengjun Wu**: software, data curation. **Shixu He**: data curation, software. **Yanling Long**: data curation. **Qiuxia Liang**: data curation. **Xionglong Tu**: data curation. **Suqiu Yao**: data curation. **Jian Wang**: conceptualization, investigation, methodology, validation, project administration, writing–original draft, writing–review and editing, resources. **Mingfang He**: conceptualization, methodology, validation, investigation, writing–original draft, writing–review and editing, project administration, resources.

## Ethics Statement

The study design was approved by the Medical and Health Research Ethics Committee of Guangyuan Central Hospital, China. All methods were carried out in accordance with relevant guidelines and regulations.

## Conflicts of interest

The authors declare no conflicts of interest.

### Peer Review

The peer review history for this article is available at https://publons.com/publon/10.1002/brb3.70147.

## Data Availability

The datasets used and/or analyzed during the current study are available from the corresponding author on reasonable request.
